# Immunogenicity and safety of BNT162b2 COVID‐19 vaccine in a chronic lymphocytic leukaemia patient

**DOI:** 10.1111/jcmm.16565

**Published:** 2021-05-25

**Authors:** Chiara Agrati, Concetta Castilletti, Alessandra Sacchi, Francesca Colavita, Maria Rosaria Capobianchi, Vincenzo Puro, Emanuele Nicastri, Giuseppe Ippolito, Michele Bibas

**Affiliations:** ^1^ National Institute for Infectious Diseases “Lazzaro Spallanzani” IRCSS Rome Italy

## CONFLICT OF INTEREST

The authors declare no competing financial interests.

## AUTHOR CONTRIBUTIONS

**Chiara Agrati:** Conceptualization (equal); Writing‐review & editing (equal). **Concetta Castilletti:** Conceptualization (equal). **Alessandra** **Sacchi:** Data curation (equal). **Francesca** **Colavita:** Data curation (equal). **Maria Rosaria** **Capobianchi:** Data curation (equal). **Vincenzo Puro:** Data curation (equal). **Emanuele** **Nicastri:** Data curation (equal). **Giuseppe** **Ippolito:** Data curation (equal). **Michele** **Bibas:** Conceptualization (equal); Writing‐review & editing (equal).

To the editor,

Up to date, more than 310 million doses of coronavirus vaccines have been administered around the world. Despite these large numbers, there are no data describing the immunogenicity and safety of commercially available vaccines against SARS‐CoV‐2 in patients with haematologic malignancies.

In particular, no clinical trials of a coronavirus disease‐19 (COVID‐19) vaccine have enrolled patients with chronic lymphocytic leukaemia (CLL), and there is not even a single report describing the capability of CLL patients to generate humoral and cellular response against COVID‐19 after vaccination.

This is very important being CLL the most common type of leukaemia in developed countries, with an age‐adjusted incidence of 4‐5 per 100.00 population.

More, CLL represents the paradigm of neoplastic disease with immunodeficiency and increased susceptibility to infections is seen from the time of diagnosis, related to quantitative and qualitative defects within the innate and adaptive immune response.^1^

Infections are the major cause of death in 30%‐50% of persons with CLL, so patients with CLL are supposed to be at high‐risk COVID‐19.

Recently, two multicentre studies have reported a high morbidity and mortality rate in CLL patients with COVID‐19, both in treated and in ‘watch and wait’ group.^2^, ^3^ Further, the development of a serologic response after SARS‐CoV‐2 infection is compromised in CLL, as one study reported that nearly only one‐third of patients develop detectable immunoglobulin G (IgG) antibodies after a median of 2 months after infection.^4^

Based on the documented lower immune response to other vaccines,^5^, ^6^ there are theoretical concerns about the capability of CLL patients to generate a fully protective immune response to SARS‐CoV‐2 vaccine.

We wanted then to answer the question if a CLL patient could mimic the robust immune response to the BNT162b2 vaccine seen in the immunocompetent population.

Here, we describe, to our knowledge, the first report of the safety and immunogenicity of BNT162b2^7^ vaccine in a patient with untreated CLL, compared with a group of 10 age‐matched healthy healthcare workers (HCWs). Our patient, a 54 year‐old physician with a five years history of treatment‐naïve CLL Stage O (according to modified Rai stage system), involved in COVID‐19 clinical management, underwent voluntary COVID‐19 vaccination after counselling in late December 2020.

**FIGURE 1:**
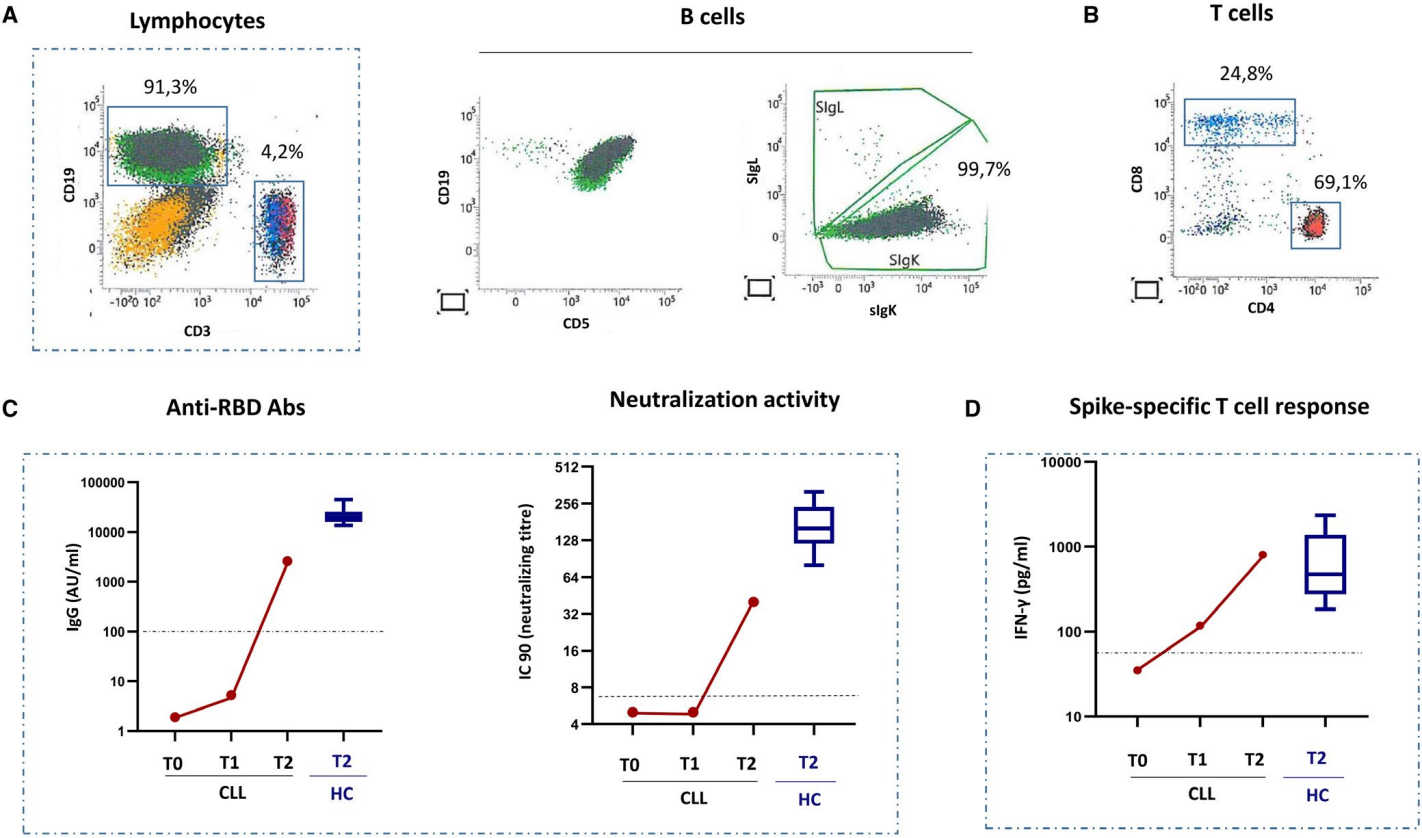
Humoral and cell‐mediated immune response to BNT162b2 vaccine in a CLL patient A, The characterization of peripheral blood mononuclear cells (PBMC) performed by flow cytometry shows the frequency and phenotype of B (CD19+) and T (CD3+) cells from the subject with CLL. B cells expressing IgK and CD5 represent the large majority of circulating PBMC, confirming a diagnosis of CLL. The frequency of T cells was low but CD4/CD8 T cell ratio was in the normal range. B,C, A significant increase in anti‐RBD IgG and in the neutralization titres was observed 2 weeks after boost (T2), although at lower level than HCWs. VNT (viral neutralization titre).D, Spike‐specific T cell response was detected before the boost (T1) and strongly increased at T2, reaching level similar to HCW. Data from HCWs (n = 10) are shown as boxer and whiskers plot (median, 25th and 75th percentile)

At the time of first dose, he had monoclonal B lymphocytosis (25 × 10^9^/L) in peripheral blood confirmed by flow cytometry as typical CLL (Figure 1A), with near‐normal gammaglobulin value (9%) and no neutropenia or associated comorbidities. T cell frequency was very low (4.2% of circulating lymphocytes) but showed a normal CD4/CD8 T cell ratio (Figure 1A).

A longitudinal analysis of humoral and cell‐mediated immune response was performed as follows: Peripheral blood was collected in the CLL subject before (T0) vaccination, after 3 weeks from the 1st dose (T1) and after 2 weeks from the 2nd dose (T2). A group of males, age‐matched healthcare workers (HCWs) (n = 10), was enrolled as a control group.

The anti‐RBD IgG was evaluated with SARS‐CoV‐2‐specific anti‐RBD Abs and quantified by CMIA (SARS‐CoV‐2 IgG II Quant, Abbott, USA). Finally, the neutralization titre was performed considering the highest serum dilution inhibiting at least 90% of the cytopathic effect (CPE) and indicated as the neutralization titre and expressed as the reciprocal of serum dilution (MNA_90_). To analyse S‐specific T cell response, heparin whole blood was stimulated for 20 hours with a pool of commercially available peptides spanning the Spike protein, and conditioned plasma was collected for IFN‐gamma quantification by ELISA assay (R&D system). To verify the T cell immunocompetence, a superantigen (SEB, 200 ng/mL, SIGMA Aldrich) was used as a positive control in each assay.

In this CLL patient, the BNT162b2 vaccination elicited a robust production of anti‐RBD IgG, with SARS‐CoV‐2 neutralization titres increasing 2 weeks after the second dose although at levels lower than HCWs. (Figure 1B). This vaccine also generated spike‐specific T cells, with interferon production reaching level similar to other HCWs. (Figure 1C). Regarding safety concerns, no specific local or systemic adverse events were noticed in the CLL patient or in the control HCWs group.

These data show the safety and immunogenicity of BNT162b2 vaccine in a stage O untreated CLL patient confirming the capability to generate humoral and cellular response against COVID‐19 after vaccination.

Finally, it is imperative to generate data to define efficacy and persistence of the immune response in similar untreated patients and in those receiving immunosuppressive therapy. CLL community is a large and heterogeneous population of patients and many of them are waiting to know when and how they can receive safely a COVID‐19 vaccine and if this strategy can significantly reduce the risk for SARS‐CoV‐2 infection.
